# Investigating the Effects of Transdiagnostic Processes on Anxiety and Depression Symptoms in Autistic Young People: the Mediating Role of Emotion Dysregulation

**DOI:** 10.1007/s10803-024-06246-0

**Published:** 2024-03-04

**Authors:** Georgina L. Barnes, Ann Ozsivadjian, Gillian Baird, Michael Absoud, Matthew J. Hollocks

**Affiliations:** 1 South London & Maudsley NHS Foundation Trust, Bethlem Royal Hospital, London, UK; 2 Department of Child & Adolescent Psychiatry, Institute of Psychiatry, Psychology & Neuroscience, King’s College London, London, UK; 3 Newcomen Centre, Evelina London Children’s Hospital, Guy’s & St Thomas’ NHS Foundation Trust, London, UK; 4 Department of Women and Children’s Health, School of Life Course Sciences, Faculty of Life Sciences and Medicine, King’s College London, London, UK

**Keywords:** Child, Autism, Emotion dysregulation, Depression, Anxiety

## Abstract

Internalising symptoms are elevated in autism compared to the general population. Few studies have investigated emotional dysregulation (ED) as a potential mediator between specific transdiagnostic processes and anxiety and depression symptoms in autistic youth. In a sample of 94 autistic young people aged 5–18 years referred to a specialist clinic for an autism evaluation, we tested the effects of ED as a mediator between cognitive inflexibility (CI), intolerance of uncertainty (IU) and alexithymia with anxiety and depression symptoms, using structural equation modelling. Effect sizes were compared to a non-autistic comparison group (n = 84). CI and alexithymia did not significantly predict depression symptoms in autistic young people, directly nor via ED. Relationships between CI/alexithymia and depression were fully mediated by ED in the non-autistic sample. There was a direct effect of CI on anxiety in the non-autistic group but not in those with a diagnosis. IU predicted depression symptoms in the autism group; and ED mediated this relationship only in those who did not receive a diagnosis. IU directly predicted anxiety in both groups and this relationship did not occur via ED. The finding of a direct pathway from IU to anxiety and depression in autistic youth is consistent with the literature. The finding that CI did not predict anxiety or depression in those with autism is novel, as was the finding that ED mediated relationships between alexithymia and anxiety/depression symptoms in both samples. The findings may have important implications for the delivery of psychological interventions for autistic youth.

## Introduction

Autism is a neurodevelopmental condition characterized by differences in social communication and social relating, restricted and repetitive behaviours and sensory processing (American Psychological Association, 2013). Autistic children and adolescents frequently experience high rates of internalising difficulties, of which anxiety is reliably found to be the most common mental health problem reported (Lau et al., 2020). Rates of low mood and depression are also consistently higher in autistic young people compared to the general population (Bougeard et al., 2021; Hollocks et al., 2022a, 2022b).

To date, evidence suggests that a range of developmental and socio-environmental factors may influence elevated rates of mental health difficulties in autistic youth (Mukherjee & Beresford, 2023). However, the specific roles of individual characteristics remain under investigation An emerging body of research highlights a central role for emotion regulation difficulties in pathways to anxiety and depression in autism. Emotion regulation (ER) is a complex process, involving a range of biological, cognitive, affectual and context-related factors. Influential theories of ER have conceptualised the term as an individual’s intentional and automatic attempts to manage changes in arousal and affect, which includes the application of internal (acquired) and external (environmental) strategies (Eisenberg, 2000; Gross, 1999). In the adult general population literature, disruptions to ER capacities are central to theories of how anxiety and depression manifest and are maintained (Hofman, 2014; Werner-Seidler et al., 2013).

Difficulty regulating affective experiences, often referred to as emotion dysregulation (ED), is found to be substantially elevated in autism compared to the general population (Connor et al., 2021). Even in the first few years of life, autistic children are found to be less easily soothed and use fewer adaptive ER strategies compared to their neurotypical peers (Cibralic et al., 2019). Reduced ER capacities in autistic young people are hypothesised to stem from a range of socio-cognitive, physiological and neural processes that are understood to be associated features of autism, including differences in cognitive processing (e.g. executive functioning, abstraction,,self-awareness), reduced emotional language, and sensitivities to environmental change and/or stimulation (Mazefsky, 2015; Mazefsky & White,^2014^). Understanding ED and associations with anxiety and mood symptoms in autistic youth is of clinical value for several reasons. First, the top priorities for treatment trials involving parents of younger autistic children are related to ED. Most psychological research has focused on the cognitive-behavioural treatment of anxiety, which shows efficacy for autistic children (Sharma et al., 2021); however, results are variable and effect sizes lower than in non-autistic youth. There has been less focus on other common problems related to ED, including depression. Across the lifespan, ED commonly presents as frequent/long-lasting negative emotions and/or social withdrawal. In older adolescents, this has been found to manifest as persistent rumination, intense reactions to social rejection and continued reliance on parents for self-regulation (Beck et al., 2020). Thus, focusing on the core processes that are theorised to underlie a range of emotional and behavioural difficulties in autism may have a broader and more sustained clinical impact compared to research that is focused solely on the secondary challenges arising from ED.

There have been various approaches to conceptualising ED within the autism literature. This has involved broad measurement of the degree of dysregulation as well as specific emotion regulation strategies/abilities, including cognitive processes relating to voluntary/involuntary engagement (e.g., problem solving vs. rumination) and disengagement (e.g., avoidance vs. numbing) (Khor et al., 2014; Conner & White, 2018; Charlton et al., 2020). New conceptual models have recently been introduced to include both the experience and regulation of emotion, as it is proposed that these two concepts intersect when measured by questionnaires (Day et al., 2022). For example, the Emotion Dysregulation Inventory (EDI; Mazefsky et al., 2018, 2021) has been developed and validated in autistic samples to provide a norm-referenced measure of the degree of dysregulation, as rated by parents/carers The EDI yields two subscales: Reactivity and Dysphoria. Reactivity refers to a state of rapidly escalating, intense and sustained negative affect, and dysphoria captures an attenuated state of general unease and poor upregulation of positive affect (Day et al., 2022). A previous large-scale study using the EDI found that clinically impairing ED was two to four times more common in autistic young people compared to non-autistic youth aged 6–17 years, after controlling for demographic differences and ADHD-related symptoms (Conner et al., 2021).

Several other key overlapping transdiagnostic processes have been linked to anxiety and depression symptoms in autistic populations. Frequently researched processes include intolerance of uncertainty, cognitive inflexibility and alexithymia. Intolerance of uncertainty (IU) has been conceptualised as a tendency to respond adversely, on an emotional, cognitive, and behavioural level, to uncertain situations and events (Buhr & Dugas, 2009). IU has been implicated in anxiety disorders and depression both in the general population (Carleton et al., 2012; Osmanağaoğlu et al., 2018) and in autistic children and adolescents (Jenkinson et al., 2020). Cognitive inflexibility (CI) has broadly been defined as the ability to flexibly adjust behaviour, thoughts or beliefs to the demands of a changing environment (e.g., Armbruster et al., 2012) and more narrowly applied to attentional set and task shifting (Dajani & Uddin, 2015). CI has been linked to flexible problem solving and transition between activities in autistic individuals (Uddin, 2021) and associated with anxiety and depression in autistic youth (Hollocks et al., 2019) and adult general population samples (Gabrys et al., 2018; Wang et al., 2019). Lastly, alexithymia refers to difficulties recognising, labelling, and processing emotions and cognitively mapping feeling states onto bodily responses (Taylor, 2000). The prevalence of alexithymia in the general population is estimated to be up to 19% and 50% in autistic individuals (Hemming et al., 2019; Kinnaird et al., 2019). Alexithymia is implicated in a range of mental health difficulties in autistic youth, including depression and anxiety (Milosavljevic et al., 2016; Oakley et al., 2022).

Research on the potential interactions between CI, IU, alexithymia and ED in pathways to mental health symptoms in autism have yielded promising results but require further investigation. It remains unclear to what extent the contribution of these transdiagnostic processes are unique to autism, or whether they contribute to anxiety and depression symptoms in non-autistic young people, but with the strength of associations being more robust in autistic individuals compared to those without a diagnosis (Boulter et al., 2014; Griffin et al., 2016; Uddin, 2021). Thus, further research is needed to compare the size of these effects in autistic and non-autistic young people, and to explore the role of ED in these pathways.

In a recent study using the current sample (Ozsivadijan et al., 2021) we found a direct effect of IU on internalising symptoms in autistic young people, with CI and alexithymia predicting internalising difficulties via IU only. This highlights the importance of testing potential cognitive, emotional and behavioural mediators thought to underlie mental health symptoms in autistic people. However, in our previous study we did not investigate specific types of internalising symptoms (i.e., anxiety vs. depression), nor did we study the role of ED in pathways to internalising difficulties. We also did not use a clinical control group to compare the size of effects between autistic and non-autistic young people. Whilst we recognise that CI, IU, alexithymia and ED are multi-factorial, overlapping constructs, it is necessary to tease these relationships apart to further our understanding of their effects on anxiety and depression symptoms in autism.. This may support the development of more effective and targeted interventions for autistic children and adolescents with co-occurring mental health difficulties.

The aims of this study were therefore to;

(1) Investigate associations between key transdiagnostic processes (IU, CI, Alexithymia) and anxiety and depression symptoms in autistic young people.

(2) Explore whether ED was a mediator of these relationships.

(3) Compare the size of effects with a non-autistic comparison group to understand whether associations are specific to having an autism diagnosis.

## Methods

### Participants and Procedure

One hundred and seventy-five young people aged 5–18 years (M = 10.6 years, SD = 3.1) and their parents were included in the study. The majority of participants (72%, n = 126) were male.

All young people included in the current study were referred to a UK specialist neurodevelopmental service for a comprehensive autism assessment between January 2015 and January 2018. Young people were accepted for a full multi-disciplinary autism evaluation following triage from an experienced clinician using probability bands on the ASD component of the Development and Wellbeing Assessment (see Measures section below) and Social Aptitudes Scale score (a measure of current social functioning included in the DAWBA).

ASD diagnoses were arrived at via a comprehensive multidisciplinary assessment and clinical consensus based on DSM-V criteria (APA, 2013). Gold-standard assessment tools were completed for every young person, including the Autism Diagnostic Interview – Revised (Lord et al., 1994) and the Autism Diagnostic Observation Schedule; (Lord et al., 2000). Families who were seen for a diagnostic assessment were also asked to complete a range of questionnaires (see following section) as part of routine clinical evaluation. All parents/carers of children under 16 years old provided consent and young people over 16 years old provided informed consent to participate in the study. NHS internal approval for anonymised use of clinical data was provided (GSTFT service evaluation number 7714). As this was a secondary data analysis, additional ethical approval was not required (see Ozsivadijan et al., 2021).

Of the 175 young people referred during this period, 94 children (54%) received a diagnosis of autism and 81 (46%) did not. Young people who did not meet the threshold for an autism spectrum diagnosis following the comprehensive assessment were treated as the control group for this study with which to compare the models. Cognitive assessment data was available for 34 autistic young people (FSIQ range = 63–122) and 36 non-autistic children (FSIQ range = 52–145). All demographic and clinical information is presented in Table 1. The primary and secondary diagnoses for both groups (other than autism) are reported in Table 2. There were moderate to large correlations between all key variables of interest (*r* range = 0.19–0.71, *p* < 0.05). See Appendix A for the univariate correlations between the key variables.

**Table 1:** Participant demographic and clinical characteristics

Variable	Autistic (n = 94)		Non-autistic (n = 81)		Total (n = 175)		F/X2 (*p*)
	M	SD	M	SD	M	SD	
Age	11.1 (5–18)	3.2	10.1 (5–17)	2.8	10.6 (5–18)	3.1	0.54 (0.5)
Sex (n male–female)	71–23	–	55–26	–	126–49	–	1.3 (0.3)
ASD symptom score (DAWBA)	26.0	1.9	21.5	2.0	23.6	1.4	2.5 (0.11)
IQ	95.4 (63–122)	2.6	94.7 (52–145)	3.0	95.0 (52–145)	6.7	0.03 (0.9)
RCADS anxiety total^a^	66.3 (34–80)	13.0	66.5 (40–80)	12.6	66.4 (34–80)	12.8	0.01 (0.9)
RCADS depression total^a^	68.3 (5–80)	13.7	69.0 (41–80)	10.4	68.6 (5–80)	12.2	0.10 (0.8)
IU^b^	42.4 (16–60)	11.3	38.9 (12–56)	11.4	40.9 (12–60)	11.4	3.3 (0.07)
CI^b^	53.7 (12–84)	16.9	46.8 (6–77)	18.6	50.4 (6–84)	18.0	6.0 (0.01)*
Alexithymia^b^	27.1 (1–42)	9.4	21.7 (3–40)	9.8	24.7 (1–42)	9.9	11.6 (0.00)*
ED reactivity^b^	53.2 (5–92)	22.7	49.2 (2–93)	25.9	51.5 (2–93)	24.1	0.8 (3.6)
ED dysphoria^b^	8.8 (0–24)	5.5	8.4 (0–24)	6.3	8.6 (0–24)	5.8	0.2 (0.7)

Measures were scored on the following scales: IU (0–60); CI (0–87); RCADS sub-scales (0–80); Alexithymia (0–42); ED reactivity (0–95); ED dysphoria (0–35)

Key: *RCADS* Revised Child Anxiety & Depression Scale, *IU* Intolerance of uncertainty, *CI* cognitive inflexibility, *ED* emotion dysregulation

^a^T-scores, ^b^Raw scores

**Table 2:** Diagnostic characteristics for the sample

Additional psychiatric diagnoses*	Autistic (n = 94) *N* (%)	Non-autistic (n = 81) (%)
Intellectual disability	13 (14%)	8 (10%)
Communication disorder	34 (36%)	35 (43%)
ADHD	23 (24%)	20 (25%)
Specific learning disorder	8 (9%)	8 (10%)
Movement disorder	10 (11%)	13 (16%)
Other neurodevelopmental condition	10 (11%)	4 (5%)
Anxiety disorder	51 (54%)	32 (40%)
Mood disorder	13 (14%)	10 (12%)
ODD/conduct	35 (37%)	27 (33%)
OCD	6 (6%)	8 (10%)
Eating/feeding disorder	8 (9%)	8 (10%)
ADHD features	10 (11%)	3 (4%)

^*^Several children had more than one co-occurring diagnosis (1 n = 167; 2 n = 133; 3 n = 69; 4 n = 24)

### Measures

#### Autism Symptoms

Following referral and acceptance to the service for an autism evaluation, all parents/carers completed the ASD module of the Development and Well-Being Assessment (DAWBA; Ford et al., 2013) online as part of the initial screening process. This module includes information required to diagnose autism, and places children in one of six probability bands indicating the percentage of children expected to receive an autism spectrum diagnosis, estimated from epidemiological samples (Goodman et al., 2011): ‘*Very low’* (< 0.1%), ‘*Low’* (1%), ‘*Low’* (3%), ‘*Moderate’* (20%), ‘*50/50’* (50%), ‘High’ (> 80%). A quantitative measure of autism symptoms was created by totalling scores from parent/carer ratings on all closed questions to the ASD module of the DAWBA, to generate a total impairment score. This procedure has been used in previous clinical research (see McEwen et al., 2016). In the current study, this measure was included as a covariate in all analyses to control for possible inter-relationships between the transdiagnostic processes, ED and internalising symptoms.

#### The Revised Child Anxiety and Depression Scale, RCADS (Chorpita et al., 2005)

The caregiver-reported RCADS (RCADS-P) is a 47-item informant-based measure for anxiety and depression symptoms in children aged 8–18 years. Items are rated on a four-point scale (0 = never, 3 = always) to provide a total raw score and six subscale scores (separation anxiety, generalised anxiety, panic, social phobia, obsessions/compulsions and depression). T-scores are also generated to provide an index of symptom severity (< 65 = Below Clinical Threshold; 65–69 = Borderline; > 70 = Above Clinical Threshold). The RCADS-P has been found to have good internal consistency and acceptable convergent and divergent validity in autistic youth (Kaat & Lecavalier, 2015). Total Anxiety and Depression T-scores were used in this study.

#### Alexithymia

The Children’s Alexithymia Measure, CAM (Way et al., 2010), is a 14-item parent-reported questionnaire measuring alexithymia in young people. Items are rated on a four-point scale (0 = almost never, 3 = almost always) providing a total raw score from 0 to 42, with higher scores indicating higher levels of alexithymia. The CAM shows good internal reliability (α = 0.92) and concurrent validity in samples of autistic youth (Griffin et al., 2016) and in the current sample (Ozsivadijan et al., 2021). For this analysis, the total score was used.

#### Intolerance of Uncertainty

The parent version of the Intolerance of Uncertainty Scale (IUS) is a 12-item caregiver-rated questionnaire assessing intolerance of uncertainty, which was developed from the original 27-item IUS (Freeston et al., 1994). It is rated on a five-point scale (0 = not at all characteristic of my child, 5 = entirely characteristic of my child) providing a total raw score from 0 to 60 (Carleton et al., 2007), with higher scores indicating greater intolerance of uncertainty. In an autistic youth sample, Boulter et al. (2014) demonstrated that the IUS had excellent internal consistency (α = 0.78). For the current analysis, the total score was used.

#### Cognitive Inflexibility

The Flexibility Scale-Revised, FS-R (Strang et al., 2017), is a 29-item parent-rated questionnaire measuring the extent of their child’s day-to-day cognitive flexibility. Items are rated on a four-point scale (0 = not at all, 3 = aways) to provide a total raw score from 0 to 87. Example items include “*perfectionistic; intolerant of error or small deviations*” and “*generally rigid or insistent*”. The FS-R has shown good divergent and convergent validity with other comparative measures in a samples of autistic youth (Strang et al., 2017). For the current study, the total score was used.

#### Emotion Dysregulation

The Emotion Dysregulation Inventory, EDI (Mazefsky et al., 2018) is a 13-item caregiver-report questionnaire designed to capture ED. Items are rated on a five-point scale (0 = not at all, 4 = very severe). The EDI has two sub-scales, reflecting factors for Reactivity (7 items) and Dysphoria (6 items). Reactivity items relate to rapidly escalating, intense, and poorly regulated negative affect. Dysphoria items relate to poor upregulation of positive affect. Although originally developed to improve measurement of ED in autistic young people, this measure demonstrates high levels of reliability in non-autistic youth. In a general population sample of children and adolescents aged 6 to 17 years, Mazefsky et al. (2021) demonstrated that the EDI has retained good convergent validity with other commonly used measures of ED in child clinical and community samples and showed excellent internal consistency on the two sub-scales (α values 0.90–0.92). As the Reactivity and Dysphoria sub-scales were highly correlated in our sample (r = 0.71, p < 0.001), we created a latent ED variable by combining the raw scores on the two sub-scales. Raw EDI scores were included in the models, as age was corrected for in all analyses.

### Statistical Analyses

Analyses were conducted using STATA. First, descriptive statistics were produced and checked for normality via visual inspection (histograms, boxplots) and traditional measures of skewness and kurtosis. All other normality assumptions for the linear regressions and mediation analyses were met (normality of residuals, univariate outliers, and multicollinearity). Data was missing from several variables; this was treated as missing at random and accounted for by using full information maximum-likelihood estimation.

**Fig. 1:**
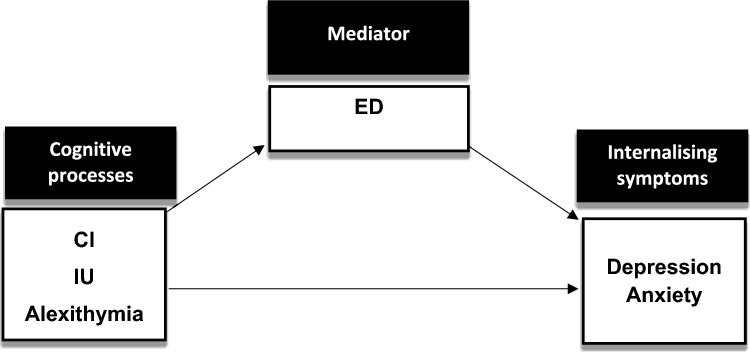
Diagram outlining the proposed SEM mediation models. *All models controlling for age, sex and autism symptoms; separate models were run for each cognitive process x internalising symptom

Next, linear regressions were employed to test associations between IU/CI/Alexithymia and ED, controlling for age, sex and autism symptoms. Structural equation models (SEM) were then estimated to investigate process-symptom links. Separate models were run for each cognitive process (CI, IU and alexithymia) to test associations with depression and anxiety, all controlling for age, sex and autism symptoms. Effect sizes were compared between the autism sample and those who did not receive an autism diagnosis. Causal mediation analysis was performed using the product of coefficients method, with the ED latent variable entered as a potential mediator in all analyses. Two key model fit indices were used to determine whether the SEM models were a good fit for the data; the model chi-square statistic and the root mean square error of approximation (RMSEA), with lower RMSEA values (< 0.06) demonstrating better model fit (Hu & Bentler, 1999). Figure 1 outlines the proposed mediation framework used to test cognitive-process symptom links, with ED as the mediating variable.

## Results

### Mechanism—Mediator Associations

There was a significant effect of CI on ED in both groups (autistic, β = 0.62, p < 0.000; non-autistic, β = 0.88, p < 0.000) when controlling for age, sex and autism symptoms. There was evidence for an effect of IU on ED in the autism sample that did not reach statistical significance (autistic, β = 0.37, p = 0.12; non-autistic, β = 0.06, p = 0.84). There was no significant effect of alexithymia on ED in either group (autistic, β = 0.14, p = 0.56; non-autistic, β = 0.34, p = 0.29). Although relationships between IU/alexithymia and ED were non-significant, these variables were included as predictors in the subsequent mediation analyses to investigate possible inter-relationships with anxiety and depression. Table 3 outlines the effects of CI, IU and alexithymia on ED by group.

**Table 3:** Linear regressions of the cognitive processes on emotional dysregulation (path a)

Predictor	Mediator
	Emotion dysregulation (Total) β (SE), *p*
Autistic group (n = 94)	
Cognitive inflexibility	**0.62 (0.17), 0.000**
Intolerance of uncertainty	0.37 (0.24), 0.12
Alexithymia	0.14 (0.23), 0.56
Non-autistic group (n = 81)	
Cognitive inflexibility	**0.88 (0.23), 0.000**
Intolerance of uncertainty	0.06 (0.28), 0.84
Alexithymia	0.34 (0.33), 0.29

Standardised Beta coefficients from ordinary least-squares linear regressions, significance: Bold = *p* < 0.05. Controlling for age, sex and autism symptoms

### Mediator—Internalising Symptom Associations

In the autistic sample, there was a significant effect of ED on depression (β = 0.29, p < 0.001) when controlling for age, sex and autism symptoms. This association was also found in the non-autistic group (β = 0.28, p < 0.001). A significant effect of ED on anxiety in both groups was also found (autistic, β = 0.30, p < 0.001; non-autistic, β = 0.30, p < 0.001).

### Mediation Models for Depression

Table 4 outlines the direct, indirect and total effects of CI, IU and alexithymia on depression by group. There was a direct effect of CI on ED in both groups when controlling for age, sex and autism symptoms (autistic group, β = 0.87; non-autistic group, β = 0.90, p values < 0.001). There was not a direct effect of CI on depression in the presence of ED (autistic, β = 0.09, non-autistic β = 0.05, *p* values > 0.05). There was an indirect (mediated) effect of CI on depression via ED in the non-autistic sample (β = 0.22, *p* < 0.0.01) that was not found in the autism group (β = 0.08, *p* > 0.59). In the final model, there was evidence of a mediated effect between CI and depression via ED in both samples; this was only statistically significant in those who did not receive an autism diagnosis (autistic β = 0.17, *p* < 0.10; non-autistic β = 0.27, *p* < 0.001). The final model was found to have good fit (*χ*^2^ = 20.3, *p* = 0.04*;* RMSEA: 0.011).

**Table 4:** Direct, indirect and total effects of CI, IU and alexithymia on depression and anxiety

Predictor	Symptom	Path c’ (Direct) β (SE), *p*	Path ab (Indirect) β (SE), *p*	Path c (Total) β (SE), *p*
Autistic (n = 94)				
CI	Depression	0.09 (0.16), 0.56	0.08 (0.14), 0.59	0.17 (0.10), 0.10
	Anxiety	0.21 (0.14), 0.15	0.08 (0.13), 0.52	**0.29 (0.09). 0.001**
IU	Depression	**0.50 (0.20), 0.01**	0.02 (0.15), 0.87	**0.47 (0.14). 0.001**
	Anxiety	**0.63 (0.16), 0.000**	0.05 (0.12), 0.67	**0.68 (0.11), 0.000**
Alexithymia	Depression	0.03 (0.23). 0.89	0.16 (0.11), 0.15	0.19 (0.20), 0.35
	Anxiety	0.002 (0.18). 0.99	**0.24 (0.11). 0.02**	0.24 (0.17). 0.16
Non-autistic (n = 81)				
CI	Depression	0.05 (0.16), 0.75	**0.22 (0.15). 0.01**	**0.27 (0.08), 0.001**
	Anxiety	**0.47 (0.17). 0.006**	0.11 (0.15), 0.48	**0.36 (0.08). 0.000**
IU	Depression	0.13 (0.14) 0.36	**0.27 (0.10). 0.01**	**0.40 (0.12), 0.001**
	Anxiety	**0.68 (0.15), 0.000**	0.05 (0.10), 0.64	**0.75 (0.12), 0.000**
Alexithymia	Depression	0.07 (0.17), 0.71	**0.37 (0.14). 0.008**	**0.44 (0.15). 0.003**
	Anxiety	0.25 (0.24), 0.28	0.20 (0.17), 0.25	**0.45 (0.17). 0.007**

Note: Emotion dysregulation was entered as a mediator in all analyses; controlling for age, sex and autism symptoms

Bold text indicates significance values *p* < 0.05

There was a direct effect of IU on ED in both groups (autistic group, β = 0.92; non-autistic group, β = 0.96, p values < 0.001).There was a direct effect of IU on depression when controlling for ED in the autistic group (β = 0.50, p = 0.01) that was not found in young people without an autism diagnosis (β = 0.13, p = 0.36). There was an indirect (mediated) effect of IU on depression via ED in the non-autistic sample that was not present in those with an autism diagnosis (autistic β = 0.02, p = 0.87; non-autistic, β = 0.27, p = 0.01). In the final model, a mediated effect between IU and depression via ED was found only in those without an autism diagnosis (autistic group, β = 0.03, p = 0.87; non-autistic group, β = 0.28, p = 0.003). The final model was found to have good fit (*χ*^2^ = 12.38, *p* = 0.34; RMSEA: 0.038).

There was a direct effect of alexithymia on ED in both groups (autistic group, β = 0.76; non-autistic group, β = 1.22, p values < 0.01). There was not a direct effect of alexithymia on depression when controlling for ED in either group (autistic group, β = 0.03; non-autistic group, β = 0.07, p values > 0.05). There was an indirect (mediated) effect of alexithymia on depression via ED in the non-autistic sample that was not present in those with an autism diagnosis (autistic group, β = 0.16, p = 0.15; non-autistic group, β = 0.37, p = 0.008). In the final model, a mediated effect between alexithymia and depression via ED was found only in those without an autism diagnosis (autistic group, β = 0.19, p = 0.35; non-autistic group, β = 0.44, p = 0.003).The final model was found to have adequate fit (*χ*^2^ = 15.58, *p* = 0.15*;* RMSEA: 0.069).

### Mediation Models for Anxiety

Table 4 outlines the direct, indirect and total effects of CI, IU and alexithymia on anxiety by group. There was a direct effect of CI on ED in both groups when controlling for age, sex and autism symptoms in the SEM models (autistic β = 0.89; non-autistic β = 0.99, *p* values < 0.001). There was a direct effect of CI on anxiety when controlling for ED in the non-autistic group (β = 0.47, p = 0.006) that was not significant for those with an autism diagnosis (β = 0.21, p = 0.14). There was not an indirect (mediated) effect of CI on anxiety via ED in either group (autistic β = 0.08, non-autistic β = 0.11, p values > 0.05). In the final model, ED did not mediate the association between CI and anxiety in either group (autistic β = 0.09, non-autistic β = 0.01, p values > 0.05). The final model had adequate fit (*χ*^2^ = 19.63, *p* = 0.05; RMSEA: 0.095).

There was a direct effect of IU on ED in both groups (autistic β = 0.97, non-autistic β = 0.99, p values < 0.000). There was a direct effect of IU on anxiety in both groups when controlling for ED (autistic β = 0.63, non-autistic β = 0.70, p values < 0.000). There was not an indirect (mediated) effect of IU on anxiety via ED in either group (autistic β = 0.05, non-autistic β = 0.05, p values > 0.05). In the final model, ED did not mediate the association between IU and anxiety in either group (autistic β = 0.05, non-autistic β = 0.04, p values > 0.05). The final model was found to have good fit (*χ*^2^ = 12.48, *p* = 0.32; RMSEA: 0.039).

There was a direct effect of alexithymia on ED in both groups (autistic β = 0.77, non-autistic β = 1.27, p values < 0.01). There was not a direct effect of alexithymia on anxiety in either group, when controlling for ED (autistic β = 0.002, non-autistic β = 0.25, p values > 0.05). There was an indirect (mediated) effect of alexithymia on anxiety via ED in the autism group (β = 0.24, p = 0.02) that was not present in young people without a diagnosis (non-autistic β = 0.19, p = 0.25). In the final model, ED mediated the association between alexithymia and anxiety in the autistic group only (autistic β = 0.32, p = 0.006; non-autistic β = 0.16, p = 0.24). The final model was found to have good fit (*χ*^2^ = 14.36, *p* = 0.02; RMSEA: 0.059).

## Discussion

To the best of our knowledge, this is the first study to have investigated the interplay between cognitive processes, ED and internalising symptoms in autistic youth, and compared these effects with a non-autistic sample. We found pathways from IU to depression and anxiety in both groups, however IU-depression links were explained by ED only in those without an autism diagnosis. There was evidence of direct and indirect pathways from CI to depression and anxiety that were only significant in the non-autistic sample. Lastly, alexithymia was not directly associated with anxiety/depression in either sample, however there was evidence for a role of ED in anxiety for autistic young people, and in depression for non-autistic young people. Our focus on ED as a mediator between well-studied cognitive processes and internalising symptoms in autism is novel, as is the comparison of these relationships in a sample of autistic and non-autistic young people. The findings highlight considerations for future research and developing targeted interventions for autistic youth with co-occurring mental health symptoms.

The lack of a direct effect between CI and depression across the sample was unexpected, as several studies have found CI to be associated with self and parent-reported depression in autistic individuals (Gabrys et al., 2018; Hollocks et al., 2014, 2022a, 2022b; Lawson et al., 2015). This lack of association and inconsistencies with the wider literature may be due to several factors, such as sampling (e.g. age and/or gender differences) and the measurement of depression and ED within the models. However, the finding of an indirect association, via ED, in young people who did not receive an autism diagnosis suggests that impaired emotion regulation processes may act as an intermediary mechanism for depression symptoms in children and adolescents, which would be useful to investigate in future research.

The finding of a large, direct effect of IU on depression symptoms in autistic young people was in keeping with previous research in this area (Cai et al., 2018; Hollocks et al., 2014). However, in our study, ED did not explain IU-depression links, which is not consistent with recent work in the autism literature (Conner et al., 2022). In addition, our finding that there was not a direct association between IU and depression in non-autistic young people does not mirror previous research with general population samples (Carleton et al., 2012; McEvoy & Mahoney, 2012). Again, this could be due to sampling and measurement differences across the studies. Moreover, as the degree of ED (as captured by the EDI) did not mediate the relationship between IU and depression in our autistic sample, this possibly suggests that more specific cognitive, emotional and/or behavioural processes may play a role which requires further exploration. In terms of potential processes, rumination and thought suppression have previously been identified as mediators of the relationship between IU and depression in the adult literature (Liao & Wei, 2011; Yook et al., 2010) however to our knowledge there is limited research examining these interrelationships in autistic and/or child and adolescent samples (see Cai et al., 2018). As rumination and suppression can be conceptualised as cognitive-behavioural markers of ED, highlights the need for more focused empirical work using valid assessment tools which tap into specific types of ED. This may further our understanding of the degree of conceptual overlap and specificity amongst key theory-driven transdiagnostic processes implicated in anxiety and depression symptoms in autistic individuals.

We also found that IU was a significant, direct predictor of anxiety symptoms in both groups, which is consistent with findings from other cross-sectional studies (Hollocks et al., 2014, 2022a, 2022b; Jenkinson et al., 2020; Osmanağaoğlu et al., 2018) and provides further empirical support for IU as a key transdiagnostic factor in anxiety in autism. Contrary to emerging work in this area (e.g. Conner et al., 2020, 2022), ED did not elicit independent effects on anxiety Instead, our findings suggested that the route from IU to anxiety may be more direct. These inter-relationships remain to be tested in future studies using a wider range of valid measures of ED and more sophisticated multivariate methods to account for possible common-method variance.

We believe that these findings make a useful contribution to the field, and the study has several strengths. First, our comparison group was made up of young people who were referred and accepted for a specialist autism assessment following robust screening procedures. We found that the two samples had comparable levels of social communication difficulties at the group level. This means that we are able to more confidently infer that group differences on the variables of interest were related to diagnosis rather than the level of social impairment. Another strength is that we used a measure of ED that has been specifically developed and validated for use in autistic populations, which strengthens the reliability and validity of the data in our sample.

Limitations of the study should also be considered. First, the cross-sectional nature of the data limits the conclusions that can be drawn about the direction of relationships between the transdiagnostic processes and internalising difficulties. Second, we used a clinically selected sample of young people, so our findings may not be generalizable to the general population. Community data is valuable given that CI, IU and alexithymia, as well as difficulties with ED and internalising symptoms are continuous/dimensional in the general population. Longitudinal studies and community samples would usefully build on this work. Another potential limitation is that participants were parents/carers of school-aged children who were seeking an autism evaluation. This may not represent the full autism spectrum, including those with moderate-severe intellectual disabilities and associated high support needs.

Third, we did not have access to item-level data for some of the caregiver-rated questionnaires, which means that we were unable to calculate the internal reliability of the measures in the study participants. In addition, all variables were highly correlated which suggested shared method variance as well as a high degree of covariance among the transdiagnostic processes and ED. All our measures were parent-report and collected from the same parent/carer at a single time point. Thus, it is possible that the mediated effects found could have been due to, in part, common-method variance, and may have impacted on the validity of the findings. Lastly, the use of parent-rated measures of internalising symptoms has limitations. Although we used a well-validated measure to assess anxiety and depression symptoms across the groups, this measure is based on informant report rather than self-report or direct observation. It is possible that parents/carers may not fully recognise a young person’s internal experiences of anxiety and depression. Future studies would benefit from multi-informant ratings of internalising symptom severity, to obtain a more complete picture of the range of symptoms and behaviours present in autistic youth with co-occurring mental health difficulties.

Given these limitations, it is important that evolution of this area of autism research focuses on more rigorous experimental tests of these constructs. Further studies are needed which use a range of multi-informant measures and assess specific elements of ED at the cognitive-behavioural level. An example is the Responses to Stress Questionnaire (RSQ; Connor-Smith et al., 2000) which captures the processes of voluntary/involuntary engagement (e.g. problem solving vs. rumination) and disengagement (e.g. avoidance vs. numbing). This measure has previously been used in studies of ED in autistic adolescents and young adults (Khor et al., 2014; Conner & White, 2018; Charlton et al., 2020) although a thorough psychometric evaluation has not yet been undertaken (Beck et al., 2020). Lastly, we focused on internalising symptoms in terms of depression and anxiety, but working from a transdiagnostic framework we might expect similar patterns to be present for other symptom categories characterised by ED (e.g., self-harm, suicidal behaviours, eating difficulties). Future research could examine the transdiagnostic generalisability of our findings to other mental health outcomes in child and adult samples.

Regarding implications for clinical practice, recent research in autistic young people (Ozsivadijan et al., 2021) has indicated a link between CI and anxiety through IU. Here, we found a direct link with IU being the strongest predictor of anxiety, although it should be noted that CI and IU were strongly correlated in this sample. Nonetheless, findings indicate that IU may be related to flexibility, which in this study was found to form a direct pathway to anxiety rather than through ED. This is important for identifying potential targets for intervention, if indeed CI and IU are underlying drivers for anxiety. Therefore, whilst both are important areas for intervention, IU may be a sensible initial target within therapies for anxiety symptoms.

This study highlights that ED processes may be potentially more proximal to depression symptoms in non-autistic young people. Transdiagnostic interventions appear promising for the treatment of different internalizing difficulties in children and adolescents, with emerging preliminary evidence suggesting that transdiagnostic targets may be just as effective in improving symptom-reduction as disorder-specific treatments, while simplifying treatment planning and delivery and increasing applicability to a wider population (Rodriguez-Seijas et al., 2020). Further work is now needed to evaluate treatment approaches that target key transdiagnostic processes implicated in depression for children and young people.

## Appendix

See Table

**Table 5:** Univariate correlations between the key variables (n = 175)

	RCADS anxiety total	RCADS depression total	IU	CI	Alexithymia	ED reactivity	ED dysphoria
RCADS anxiety total	–						
RCADS depression total	0.60**	–					
IU	0.62**	0.40**	–				
CI	0.44**	0.30**	0.64**	–			
Alexithymia	0.19*	0.20*	0.47**	0.54**	–		
ED reactivity	0.24*	0.23*	0.47**	0.69**	0.47**	–	
ED dysphoria	0.40**	0.40**	0.56**	0.62**	0.44**	0.71**	–

Key: *RCADS* Revised Child Anxiety & Depression Scale, *IU* Intolerance of uncertainty, *CI* cognitive inflexibility, *ED* emotion dysregulation

^**^Correlation is significant at the 0.001 level (2-tailed), *Correlation is significant at the 0.05 level (2-tailed)

5.
